# The Synthesis and Structure of a Scandium Nitrate Hydroxy-Bridged Dimeric Complex Supported by Bipyridyl Ligands †

**DOI:** 10.3390/molecules27062024

**Published:** 2022-03-21

**Authors:** Simon A. Cotton, Paul R. Raithby, Stephanie Schiffers, Simon J. Teat, John E. Warren

**Affiliations:** 1 School of Chemistry, University of Birmingham, Edgbaston, Birmingham B15 2TT, UK; 2 Department of Chemistry, University of Bath, Bath BA2 7AY, UK; p.r.raithby@bath.ac.uk (P.R.R.); chsprr@bath.ac.uk (S.S.); 3 Advanced Light Source, Lawrence Berkeley National Laboratory, Berkeley, CA 94720, USA; sjteat@lbl.gov; 4 Department of Mechanical, Aerospace & Civil Engineering, University of Manchester, Manchester M1 3BB, UK; john.warren@manchester.ac.uk

**Keywords:** crystal structure, scandium complex, eight coordinate complex, nitrate complex, hydroxy group

## Abstract

The current discussion on whether scandium, yttrium and lanthanum should represent Group 3 in the Periodic Table or whether lutetium should replace lanthanum in the group has prompted us to further explore the structural chemistry of the Group 3 elements and compare the coordination numbers and coordination geometries adopted. The steric and electronic properties of the coordinated ligands have a major influence on the structures adopted. We report the synthesis and crystal structure determination of an unusual dinuclear scandium complex [(bipy)(NO_3_)_2_Sc(µ-OH)_2_Sc(NO_3_)_2_(bipy)] obtained by the reaction of hydrated scandium nitrate with 2,2′-bipyridyl (bipy) in either ethanol or nitromethane. The crystal structure of the complex shows that the scandium centers are eight coordinate, and the structure obtained contrasts with related complexes found in the lanthanide series [Ln(bipy)_2_(NO_3_)_3_] and [Ln(phen)_2_(NO_3_)_3_] (phen = phenanthroline) and in [M(terpy)(NO_3_)_3_] (M = Sc, Er–Lu), where these complexes are all mononuclear.

## 1. Introduction

The form and arrangement of the Periodic Table is of considerable current interest [1]. Some of this interest comes from the discussion of whether lanthanum should be classed as a Group 3 element along with scandium and yttrium [2] or whether lutetium is better suited to be a member of this group than lanthanum [3,4]. From the viewpoint of structural chemistry, the coordination number and geometry adopted by the +3 metal ion in a complex is dependent on the size of the ion, with the steric requirements of the ligands playing a secondary role; ligand field effects do not contribute to complexes of the Ln(III) ions or for Sc(III) and Y(III). For example, the smaller Sc(III) ion forms a seven coordinate aqua ion in contrast to the nine coordinate [La(H_2_O)_9_]^3+^ ion that is typical of the larger early lanthanides and the eight coordinate [M(H_2_O)_8_]^3+^ ions formed by Y(III) and the heavier actinides, which have intermediate ionic radii [5]. A recent systematic structural study of analogous complexes of Sc(III), Y(III), La(III) and Lu(III) showed that there were 29 sets of compounds where at least three of the elements form compounds with the same ligands have been identified and their crystal structures determined [6]. In 14 of the sets, the scandium and lutetium complexes have the same coordination number; but in the remaining 15 they do not. Since the ionic radii of Sc(III) and Lu(III) are quite similar (0.75 Å (6 coordinate) and 0.98 Å (8 coordinate), respectively [7]), the observation that approximately half of the cases where Sc(III) and Lu(III) form equivalent compounds indicates that the nature of the ligands in these complexes is important in determining the resultant coordination number and geometry. This has prompted us to investigate the coordination chemistry of Sc(III) complexes further looking for differences in coordination geometry and number with similar ligand sets.

For example, scandium nitrate forms a complex with 2,2′:6,2′′-terpyridine having the formula [Sc(terpy)(NO_3_)_3_] [8] very similar to [Ln(terpy)(NO_3_)_3_] (Ln, e.g., Yb, Lu) obtained with the heaviest lanthanides [9]. We thus were interested to see whether scandium nitrate formed a 2,2′-bipyridyl (bipy) complex that resembled the well-known ten coordinate [Ln(bipy)_2_(NO_3_)_3_] and [Ln(phen)_2_(NO_3_)_3_] (Ln = Y, La–Lu except Pm) [10,11,12], especially as one report of [Sc(phen)_2_(NO_3_)_3_] and one of [Sc(bipy)_2_(NO_3_)_3_] have appeared [13,14].

## 2. Results and Discussion

Using synthesis conditions similar to those used by ourselves and others for [Ln(bipy)_2_(NO_3_)_3_] (Ln = Y, La–Lu) [10,11,12], we obtained a somewhat powdery product, but by reaction of hot dilute ethanolic solutions of hydrated scandium nitrate and bipy, in a 1:2 ratio, followed by very slow cooling (immersed in a Dewar of very hot water) yielded small colorless crystals suitable for study by synchrotron single crystal X-ray diffraction methods.

The infrared spectrum of the product contains absorptions due to both the nitrate group and the 2,2′-bipyridyl ligand, and it is not always possible to distinguish them. Thus, absorptions at 1020 and 1033 cm^−1^ are probably due, respectively, to a ring breathing vibration and to ν1(A_1_) of the nitrate group. Strong bands at 1315 and 1331 cm^−1^ are assigned to a ligand vibration and to a vibration of the coordinated nitrate group, as are three absorptions at 1441, 1476 and 1537 cm^−1^. A broad, weak vibration centered upon ~3090 cm^−1^ is probably due to ν(O-H) stretching vibrations (See Supplementary Materials Figure S1a). A MALDI mass spectrum of the compound (See Figure S1b) displayed a molecular ion with a *m/z* peak at 682.94 [M – H]^+^, suggesting that a dimeric complex had formed. The X-ray single-crystal structure determination of the product identified it to be an unexpected dimeric complex, [(bipy)(NO_3_)_2_Sc(μ-OH)_2_Sc(NO_3_)_2_(bipy)]. We investigated the use of nitromethane as an alternative solvent for the reaction, following the example of Junk et al. [15], who isolated [In(bipy)_2_(NO_3_)_3_] from this medium, but the product of this reaction was identical to that obtained from ethanol.

**Figure 1:**
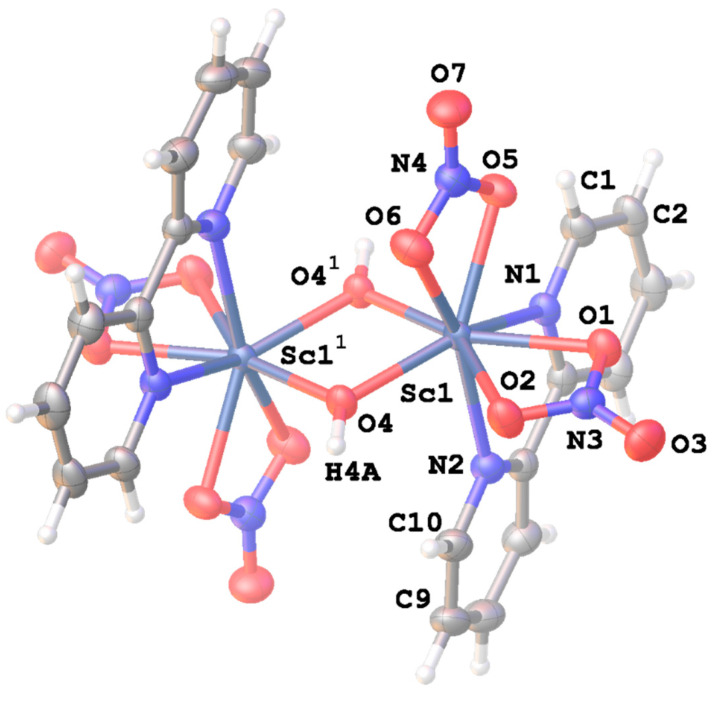
The molecular structure of the dimeric [(bipy)(NO_3_)_2_Sc(μ-OH)_2_Sc(NO_3_)_2_(bipy)] showing the atom numbering scheme. The atoms with the superscript ^1^ are related to the atoms in the asymmetric unit by the symmetry operator −x, −y, 1 − z. The displacement ellipsoids are set at 50% probability. Selected bond parameters: Bond lengths: Sc1-O1, 2.3115(17), Sc1-O2, 2.2958(18), Sc1-O4, 2.0974(15), Sc1-O4^1^, 2.0413(16), Sc1-O5, 2.2702(17), Sc1-O6, 2.2558(19), Sc1-N1, 2.3744(19), Sc1-N2, 2.3261(19), Sc1···Sc1^1^, 3.3372(9) Å; Bond angles: O2-Sc1-O1, 55.05(6), O6-Sc1-O5, 55.76(6), N2-Sc1-N1,69.09(6), O4^1^-Sc1-O4, 72.52(7), Sc1^1^-O4-Sc1, 107.48(7)°. Atoms denoted “^1^” are related by the symmetry operation −x, −y, 1 − z.

The molecular structure of [(bipy)(NO_3_)_2_Sc(μ-OH)_2_Sc(NO_3_)_2_(bipy)] is shown in Figure 1, which includes selected bond parameters (a full list of bond parameters is available in the Supplementary Materials, Tables S4–S7). The molecule sits on a crystallographic center of symmetry at the center point of the Sc_2_(μ-OH)_2_ rhombus, with one Sc atom in the crystallographic asymmetric unit. The unique scandium is eight coordinate, bound to four oxygen atoms from two bidentate nitrate groups, two nitrogen atoms from the bipyridyl ligand and two oxygen atoms from the bridging hydroxyl groups. The coordination geometry is best described as a distorted triangulated dodecahedron. The bipy ligand is essentially planar. There is a significant asymmetry in the double hydroxy bridging unit with the two independent Sc-O bonds differing by ca. 0.05 Å, as there is in the Sc-N bond lengths to the bipy ligand where the difference is also ca. 0.05 Å. There is also slight asymmetry in the bidentate coordination of the nitrates, with Sc-O (nitrate) distances falling in a range 2.2558(19) to 2.3115(17) Å, averaging 2.283 Å, which compare closely with those (2.279 Å) in eight coordinate [Sc(H_2_O)_4_(NO_3_)_2_]^+^(NO_3_)^−^·2H_2_O [16], but slightly longer than the average of 2.240 Å in [Sc(NO_3_)_3_(H_2_O)_2_][(12-crown-4)]_2_ [17]. Comparison of the average bond lengths of 2.069 Å for Sc-O(OH) and 2.283 Å for Sc-O (NO_3_) in [(bipy)(NO_3_)_2_Sc(μ-OH)_2_Sc(NO_3_)_2_(bipy)] with the corresponding values of 2.062 Å and 2.297 Å in seven coordinate [(NO_3_)(H_2_O)_3_Sc(μ-OH)_2_Sc(H_2_O)_3_(NO_3_)]^2+^ (NO_3_)^−2^ [18] shows close similarity.

The complex crystallizes in the triclinic space group *P-1* (no. 2) with half a molecule in the asymmetric unit. Adjacent molecules in the crystal are linked by a hydrogen-bond, with the bridging hydroxy group acting as the H-bond donor, and a nitrate oxygen atom on an adjacent molecule acting as the H-bond acceptor (O4-H4A···O3^1^; H4A···O3^1^ 2.29(4), O4···O3^1^ 3.174(3) Å, O4-H4A···O3^1^ 166(3)°; symmetry code (^1^) 1 − x, −y, 1 − z). The presence of the H-bonding is confirmed by a Hirshfeld analysis and the generation of fingerprint plots using CrystalExplorer 17.5 [19] shown in Figures S1 and S2 (see Supplementary Materials). In the supramolecular architecture these H-bond interactions pair up to form 12-membered rings, R2,2(12) in Etter notation [20] as illustrated in Figure S3 (see Supplementary Materials).

Isolated dimeric bis(μ-hydroxy) complexes are not uncommon for scandium, examples including [py_2_Cl_2_Sc(μ-OH)_2_ScCl_2_py2]·4py (py = pyridine) [21], [(H_2_O)_5_Sc(μ-OH)_2_Sc(H_2_O)_5_]X_4_·2H_2_O (X = Cl, Br) [22] and [(NO_3_)(H_2_O)_3_Sc(μ-OH)_2_Sc(H_2_O)_3_(NO_3_)] (NO_3_)_2_ [18], which involve six-, seven- and seven coordinate scandium centers, respectively. The higher coordination number of scandium in [(bipy)(NO_3_)2Sc(μ-OH)_2_Sc(NO_3_)_2_(bipy)] can be attributed to the presence in the coordination sphere of three bidentate ligands, with nitrate having an especially small bite angle, rather than exclusively monodentate ligands, as in these three examples.

Junk et al. [15] obtained a similar compound, [(bipy)(NO_3_)_2_In(μ-OH)_2_In(NO_3_)_2_(bipy)], on one occasion from the reaction of hydrated indium nitrate with 2,2′-bipyridyl in nitromethane. The poorly formed crystals gave a structure of rather low precision, but average In-N distances are rather shorter, at 2.25 Å, and In-OH and In-O (NO_3_) distances longer at 2.14 and 2.52 Å, respectively, than in the scandium compound. On the basis of ionic radii [7], they would be expected to be some 0.04 Å longer. The isolation of this compound indicates that hydrolysis of [M(H_2_O)_6_]^3+^ ions occurs for larger metals than scandium.

The corresponding reaction between scandium nitrate and phenanthroline in methanol results in a rather similar complex, eight coordinate [(phen)(NO_3_)_2_Sc(µ-OMe)_2_Sc(NO_3_)_2_(phen)], this time with two methoxy bridges [23]. In this compound, average bond lengths of 2.077 Å for Sc-O(OMe) and 2.287 Å for Sc-O (NO_3_) are similar to those in the bipyridyl complex.

Mononuclear species predominate in Sc(III) (aq) solutions at low pH, but there is significant hydrolysis under less acidic conditions, with [(H_2_O)_5_Sc(μ-OH)_2_Sc(H_2_O)_5_]^4+^ species predominant in the absence of coordinating counter-ions. Sc(NO_3_)_3_·5H_2_O has a solid-state structure containing [Sc(H_2_O)_4_(NO_3_)_2_]^+^ ions with bidentate nitrates and eight coordinate scandium [16]; at pH 3, it is known to hydrolyze [18] to the dimeric [(NO_3_)(H_2_O)_3_Sc(µ-OH)_2_Sc(H_2_O)_3_(NO_3_)]^2+^ (NO_3_)^−2^. The isolation of [(bipy)(NO_3_)_2_Sc(μ-OH)_2_Sc(NO_3_)_2_(bipy)] from the reaction we employed suggests that there are significant amounts of a dimer present in solutions of scandium nitrate in ethanol or nitromethane. The larger tripositive lanthanide ions are not as prone to hydrolysis as scandium, with [Ln(NO_3_)_3–x_ (solvent)*_n_*]_(3−x)_^+^ (x = 1–3) predominating in solution, resulting in mononuclear bipyridyl complexes [Ln(NO_3_)_3_(bipy)_2_] being obtained [24]. Evidently the smaller radius of the Sc^3+^ ion and greater polarizing power makes it a stronger Lewis acid than the lanthanide ions, promoting hydrolysis of the aqua ion and concomitant dimerization; this is reflected in the pKa value for Sc^3+^(aq) of 4.3, compared with the respective values of 8.5 and 7.6 for La^3+^(aq) and Lu^3+^(aq) [25], a cause of the significant differences between the chemistry of scandium and the lanthanides [6].

## 3. Materials and Methods

Hydrated scandium nitrate, 2,2′-bipyridine, and solvents were obtained as commercial products (Aldrich) and were used without purification.

A hot solution of hydrated scandium nitrate (0.10 g, 0.31 mmol) in ethanol (5 mL) was mixed with 2,2′-bipyridine (0.10 g, 0.64 mmol) in hot ethanol (25 mL) and allowed to stand with very slow cooling overnight, Colorless crystals were obtained on standing for 2 days. The reaction was repeated using nitromethane instead of ethanol as the solvent and the same reaction product was obtained.

The IR spectrum of the solid was recorded on a Nicolet Avatar 360 FTIR spectrometer. IR: ν /cm^−1^: 3624 (w), 3092 (w), 1653 (s), 1599 (sh), 1568 (sh), 1537 (m), 1511 (m), 1476 (m), 1441 (s), 1359 (w), 1331 (sh), 1315 (vs), 1296 (s), 1179 (w), 1106 (w), 1065 (sh), 1033 (sh), 1020 (s), 814 (sh), 799 (m), 767 (s), 737 (s), 651 (m), 631 (w), 619 (w). A MALDI mass spectrum was recorded on a Micromass MALDI Micro MX TOF, which displayed a molecular ion corresponding to [M − H]^+^ at 682.94.

The crystal data, data collection parameters, and structure solution and refinement details for the crystal structure of [(bipy)(NO_3_)_2_Sc(µ-OH)_2_Sc(NO_3_)_2_(bipy)] are summarized in Table S1 (Supplementary Materials). The crystal data was collected on a Bruker AXS SMART diffractometer (Madison, WI, USA), equipped with an Oxford Cryostream cooling apparatus, at Station 9.8 of the CCLRC Daresbury Laboratory, UK, using monochromatic X-ray radiation of wavelength 0.6911 Å. Structure solution was achieved by direct methods and refined by full-matrix least-squares on *F*^2^ using SHELXL-2014 [26] within the OLEX-2 suite [27], with all ordered non-hydrogen atoms assigned anisotropic displacement parameters. Hydrogen atoms attached to carbon atoms were placed in idealized positions and allowed to ride on the relevant carbon atom. The unique hydroxy hydrogen atom was located in the electron density difference map and refined freely. In the final cycles of refinement, a weighting scheme that gave a relatively flat analysis of variance was introduced and refinement continued until convergence was reached.

## 4. Conclusions

Reaction of hot dilute ethanolic solutions of hydrated scandium nitrate and bipy led to the formation of a dimeric, di-hydroxy bridge scandium complex, [(bipy)(NO_3_)_2_Sc(µ-OH)_2_Sc(NO_3_)_2_(bipy)]. An X-ray crystal structure determination of the product shows that the scandium centers are eight coordinate, and this contrasts to related complexes of slightly larger lanthanides obtained under similar reaction conditions, [Lu(bipy)_2_(NO_3_)_3_] and [Lu(phen)_2_(NO_3_)_3_] (phen = phenanthroline), which are mononuclear and have higher coordination numbers.

## Data Availability

Crystal data is available from the Cambridge Crystallographic Data Centre 12 Union Road, Cambridge CB2 1EZ, UK; fax: (+44)-1223-336-033 or e-mail: deposit@ccdc.cam.ac.uk (CCDC No: 2143609). The data may be obtained free of charge via http://www.ccdc.cam.ac.uk/conts/retrieving.html (accessed on 22 December 2021).

## Supplementary Materials

The following supporting information can be downloaded at: https://www.mdpi.com/article/10.3390/molecules27062024/s1, Table S1: Crystal data and structure refinement for bath333n; Table S2: Fractional Atomic Coordinates (×10^4^) and Equivalent Isotropic Displacement Parameters (Å^2^ × 10^3^) for bath333n. U_eq_ is defined as 1/3 of the trace of the orthogonalized U_IJ_ tensor; Table S3: Anisotropic Displacement Parameters (Å^2^ × 10^3^) for bath333n. The Anisotropic displacement factor exponent takes the form: −2π^2^[h^2^a × 2U_11_ + 2hka × b × U_12_ + …]; Table S4: Bond Lengths for bath333n; Table S5: Bond Angles for bath333n; Table S6: Hydrogen Bonds for bath333n; Table S7: Torsion Angles for bath333n; Table S8: Hydrogen Atom Coordinates (Å × 10^4^) and Isotropic Displacement Parameters (Å^2^ × 10^3^) for bath333n; Figure S1: MALDI mass spectrum and solid state IR of the complex; Figure S2: Surface plot of D-norm surface-0_3117_1_0733; Figure S3: Fingerprint plot showing the presence of hydrogen bonding; Figure S4: Packing plot showing the H-hydrogen bonding interactions and the presence of the R2,2(12) rings. Supplementary crystallographic data (cif file) has been deposited at the Cambridge Crystallographic Data Centre (CCDC No: 2143609). The data may be obtained free of charge via http://www.ccdc.cam.ac.uk/conts/retrieving.html (accessed on 22 December 2021) or from the Cambridge Crystallographic Data Centre, 12 Union Road, Cambridge CB2 1EZ, UK; fax: (+44)-1223-336-033 or e-mail: deposit@ccdc.cam.ac.uk.

## Sample Availability

A sample of the title compound is available from S.A.C.
