# Supplementary material for: The Synthesis and Structure of a Scandium Nitrate Hydroxy-Bridged Dimeric Complex Supported by Bipyridyl Ligands
Source: Molecules. 2022 Mar 21;27(6):2024. doi: 10.3390/molecules27062024 (PMC8954743; doi:10.3390/molecules27062024)
Supplement: Supplementary file 1 [file molecules-27-02024-s001.zip › molecules-1638473-supplementary.pdf]

SUPPLEMENTARY MATERIALS

The Synthesis and Structure of a Scandium Nitrate Hydroxy-Bridged Dimeric Complex Supported by Bipyridyl Ligands

Simon A. Cotton, Paul R. Raithby, Stephanie Schiffers, Simon J. Teat and John E. Warren

bath333n

Table S1 Crystal data and structure refinement for bath333n.

Identification code	bath333n
Empirical formula	$C_{20}H_{18}N_8O_{14}Sc_2$
Formula weight	684.34
Temperature/K	150.15
Crystal system	triclinic
Space group	P-1
a/Å	8.059(2)
b/Å	9.214(3)
c/Å	10.025(3)
α/°	72.191(3)
β/°	69.448(3)
γ/°	69.438(3)
Volume/Å^3	638.4(3)
Z	1
ρ_{calc}g/cm^3	1.780
μ/mm^{-1}	0.578
F(000)	348.0
Crystal size/mm^3	0.1 × 0.05 × 0.05
Radiation	synchrotron (λ = 0.6911)
2Θ range for data collection/°	6.15 to 60.146
Index ranges	-11 ≤ h ≤ 11, -13 ≤ k ≤ 13, -14 ≤ l ≤ 14
Reflections collected	7072
Independent reflections	3770 [R_{int} = 0.0325, R_{sigma} = 0.0498]
Data/restraints/parameters	3770/0/203
Goodness-of-fit on F^2	1.044
Final R indexes [I>=2σ (I)]	R_1 = 0.0534, wR_2 = 0.1609
Final R indexes [all data]	R_1 = 0.0625, wR_2 = 0.1704
Largest diff. peak/hole / e Å^{-3}	0.89/-0.51

Table S2 Fractional Atomic Coordinates (×10^4) and Equivalent Isotropic Displacement Parameters (Å^2×10^3) for bath333n. U_{eq} is defined as 1/3 of the trace of the orthogonalised U_{IJ} tensor.

Atom	*x*	*y*	*z*	U(eq)
C1	-268(3)	3019(3)	778(2)	30.8(4)
C2	-348(3)	3254(3)	-632(3)	35.9(5)
C3	754(4)	2118(3)	-1430(3)	40.1(6)
C4	1904(4)	767(3)	-797(3)	35.6(5)
C5	1915(3)	604(2)	622(2)	25.7(4)
C6	3107(3)	-804(2)	1370(2)	25.3(4)
C7	4017(3)	-2162(3)	800(3)	34.6(5)
C8	5089(3)	-3436(3)	1560(3)	40.1(5)
C9	5280(3)	-3313(3)	2822(3)	37.9(5)
C10	4330(3)	-1921(3)	3330(3)	32.4(4)
N1	854(2)	1727(2)	1407.9(19)	25.2(3)
N2	3227(2)	-686(2)	2635(2)	27.0(3)
N3	4184(2)	2238(2)	3131(2)	28.1(4)
N4	-1136(3)	3944(2)	4793(2)	29.6(4)
O1	3097(2)	2803(2)	2330.5(18)	31.5(3)
O2	3636(2)	1328(2)	4315.6(19)	35.0(4)
O3	5668(2)	2519(2)	2775(2)	40.1(4)
O4	1171(2)	-502.9(18)	5666.9(16)	26.3(3)
O5	-857(2)	3793.8(19)	3511.4(18)	33.5(4)
O6	-270(3)	2755(2)	5549(2)	36.9(4)
O7	-2156(3)	5121(2)	5244(2)	41.3(4)
Sc1	1081.6(5)	1313.3(4)	3801.5(4)	22.46(14)

Table S3 Anisotropic Displacement Parameters (Å^2×10^3) for bath333n. The Anisotropic displacement factor exponent takes the form: $-2\pi^2[h^2a^{*2}U_{11}+2hka^*b^*U_{12}+\ldots]$.

Atom	U_{11}	U_{22}	U_{33}	U_{23}	U_{13}	U_{12}
C1	32.0(10)	32.7(10)	29.2(10)	0.3(7)	-16.7(8)	-7.9(8)
C2	39.0(11)	41.1(12)	30.2(11)	7.4(9)	-20.7(9)	-15.4(10)
C3	48.7(13)	54.6(15)	24.7(10)	2.0(9)	-19.5(10)	-22.4(12)
C4	40.9(12)	46.2(12)	24.4(10)	-7.5(8)	-10.9(9)	-15.3(10)
C5	25.9(8)	31.9(9)	22.6(9)	-2.4(7)	-9.1(7)	-12.3(7)
C6	24.3(8)	28.5(9)	24.5(9)	-3.8(7)	-7.3(7)	-9.7(7)
C7	31.8(10)	38.4(12)	36.1(12)	-14.5(9)	-7.4(9)	-8.6(9)
C8	31.4(10)	32.9(11)	52.9(15)	-14.5(10)	-9.1(10)	-2.8(9)
C9	28.8(10)	31.2(11)	47.1(14)	-4.3(9)	-13.3(9)	-0.8(8)
C10	28.3(9)	33.7(10)	34.3(11)	-5.1(8)	-13.7(8)	-3.7(8)
N1	27.0(8)	28.0(8)	22.5(7)	-0.4(6)	-12.2(6)	-8.3(6)
N2	26.5(8)	28.1(8)	26.8(8)	-4.1(6)	-11.1(7)	-5.7(6)
N3	26.1(8)	29.6(8)	31.2(9)	-8.7(6)	-10.2(7)	-6.1(6)
N4	31.5(8)	28.5(8)	32.0(9)	-6.4(6)	-11.8(7)	-8.7(7)

Table S3 Anisotropic Displacement Parameters (Å^2×10^3) for bath333n. The Anisotropic displacement factor exponent takes the form: $-2\pi^2[h^2a^{*2}U_{11}+2hka^*b^*U_{12}+\ldots]$.

Atom	U_{11}	U_{22}	U_{33}	U_{23}	U_{13}	U_{12}
O1	34.6(8)	35.2(8)	27.4(7)	0.9(6)	-14.5(6)	-12.8(6)
O2	37.9(8)	36.4(8)	36.4(9)	3.1(6)	-21.2(7)	-15.0(7)
O3	30.8(8)	48.8(10)	46.7(10)	-11.3(8)	-11.2(7)	-16.2(7)
O4	28.0(7)	30.3(7)	22.6(7)	1.1(5)	-13.1(5)	-9.9(6)
O5	40.3(8)	29.7(8)	25.6(7)	-4.6(6)	-12.6(6)	-1.4(6)
O6	47.7(10)	32.7(8)	35.0(9)	-6.7(6)	-21.8(8)	-6.5(7)
O7	42.8(9)	34.9(9)	46.6(11)	-18.8(7)	-9.1(8)	-5.2(7)
Sc1	24.2(2)	23.9(2)	19.9(2)	-1.21(13)	-10.03(14)	-6.09(14)

Table S4 Bond Lengths for bath333n.

Atom	Atom	Length/Å	Atom	Atom	Length/Å
C1	C2	1.384(3)	N3	O2	1.261(3)
C1	N1	1.348(3)	N3	O3	1.219(3)
C2	C3	1.373(4)	N3	Sc1	2.719(2)
C3	C4	1.389(4)	N4	O5	1.267(3)
C4	C5	1.388(3)	N4	O6	1.261(3)
C5	C6	1.485(3)	N4	O7	1.212(3)
C5	N1	1.351(3)	N4	Sc1	2.691(2)
C6	C7	1.389(3)	O1	Sc1	2.3115(17)
C6	N2	1.344(3)	O2	Sc1	2.2958(18)
C7	C8	1.381(4)	O4	Sc1[1]	2.0413(16)
C8	C9	1.369(4)	O4	Sc1	2.0974(15)
C9	C10	1.388(3)	O5	Sc1	2.2702(17)
C10	N2	1.346(3)	O6	Sc1	2.2558(19)
N1	Sc1	2.3744(19)	Sc1	O4[1]	2.0412(16)
N2	Sc1	2.3261(19)	Sc1	Sc1[1]	3.3372(9)
N3	O1	1.265(3)			

[1]-X,-Y,1-Z

Table S5 Bond Angles for bath333n.

Atom	Atom	Atom	Angle/°	Atom	Atom	Atom	Angle/°
N1	C1	C2	123.1(2)	N4	Sc1	Sc1[1]	98.96(5)
C3	C2	C1	118.7(2)	O1	Sc1	N1	74.78(6)
C2	C3	C4	119.2(2)	O1	Sc1	N2	83.98(7)
C5	C4	C3	119.2(2)	O1	Sc1	N3	27.64(6)
C4	C5	C6	122.2(2)	O1	Sc1	N4	87.26(6)
N1	C5	C4	122.1(2)	O1	Sc1	Sc1[1]	168.01(4)
N1	C5	C6	115.70(17)	O2	Sc1	N1	124.04(6)

Table S5 Bond Angles for bath333n.

Atom	Atom	Atom	Angle/°	Atom	Atom	Atom	Angle/°
C7	C6	C5	121.7(2)	O2	Sc1	N2	81.61(7)
N2	C6	C5	115.82(18)	O2	Sc1	N3	27.51(6)
N2	C6	C7	122.5(2)	O2	Sc1	N4	95.65(7)
C8	C7	C6	118.7(2)	O2	Sc1	O1	55.05(6)
C9	C8	C7	119.5(2)	O2	Sc1	Sc1[1]	113.80(5)
C8	C9	C10	118.7(2)	O4	Sc1	N1	138.96(6)
N2	C10	C9	122.7(2)	O4[1]	Sc1	N1	81.94(6)
C1	N1	C5	117.73(18)	O4	Sc1	N2	82.84(7)
C1	N1	Sc1	124.05(15)	O4[1]	Sc1	N2	97.61(7)
C5	N1	Sc1	118.22(13)	O4	Sc1	N3	105.20(6)
C6	N2	C10	117.74(19)	O4[1]	Sc1	N3	177.09(6)
C6	N2	Sc1	119.45(14)	O4	Sc1	N4	105.27(7)
C10	N2	Sc1	121.27(15)	O4[1]	Sc1	N4	89.09(6)
O1	N3	Sc1	57.95(10)	O4[1]	Sc1	O1	154.43(6)
O2	N3	O1	114.88(18)	O4	Sc1	O1	132.73(6)
O2	N3	Sc1	57.22(11)	O4[1]	Sc1	O2	150.51(6)
O3	N3	O1	122.8(2)	O4	Sc1	O2	78.16(6)
O3	N3	O2	122.3(2)	O4[1]	Sc1	O4	72.52(7)
O3	N3	Sc1	173.56(16)	O4	Sc1	O5	130.34(6)
O5	N4	Sc1	57.20(10)	O4[1]	Sc1	O5	86.79(7)
O6	N4	O5	113.72(18)	O4[1]	Sc1	O6	91.81(7)
O6	N4	Sc1	56.52(11)	O4	Sc1	O6	79.76(7)
O7	N4	O5	122.7(2)	O4	Sc1	Sc1[1]	35.69(4)
O7	N4	O6	123.6(2)	O4[1]	Sc1	Sc1[1]	36.83(4)
O7	N4	Sc1	179.04(15)	O5	Sc1	N1	77.97(6)
N3	O1	Sc1	94.42(12)	O5	Sc1	N2	145.68(6)
N3	O2	Sc1	95.27(12)	O5	Sc1	N3	96.10(7)
Sc1[1]	O4	Sc1	107.48(7)	O5	Sc1	N4	27.97(6)
N4	O5	Sc1	94.83(12)	O5	Sc1	O1	78.24(7)
N4	O6	Sc1	95.69(13)	O5	Sc1	O2	110.48(7)
N1	Sc1	N3	98.99(6)	O5	Sc1	Sc1[1]	111.88(5)
N1	Sc1	N4	105.89(6)	O6	Sc1	N1	133.63(6)
N1	Sc1	Sc1[1]	112.84(5)	O6	Sc1	N2	156.71(7)
N2	Sc1	N1	69.09(6)	O6	Sc1	N3	89.53(7)
N2	Sc1	N3	80.20(6)	O6	Sc1	N4	27.79(6)
N2	Sc1	N4	170.81(6)	O6	Sc1	O1	96.58(7)
N2	Sc1	Sc1[1]	90.16(5)	O6	Sc1	O2	79.77(7)
N3	Sc1	Sc1[1]	140.86(4)	O6	Sc1	O5	55.76(6)
N4	Sc1	N3	93.29(6)	O6	Sc1	Sc1[1]	84.70(5)

[1]-X,-Y,1-Z

Table S6 Hydrogen Bonds for bath333n.

D	H	A	d(D-H)/Å	d(H-A)/Å	d(D-A)/Å	D-H-A/°
C1	H1	O5	0.95	2.24	2.879(3)	123.7

Table S7 Torsion Angles for bath333n.

A	B	C	D	Angle/˚	A	B	C	D	Angle/˚
C1	C2	C3	C4	0.4(4)	C7	C6	N2	Sc1	-163.51(16)
C2	C1	N1	C5	-1.3(3)	C7	C8	C9	C10	2.4(4)
C2	C1	N1	Sc1	178.45(17)	C8	C9	C10	N2	0.0(4)
C2	C3	C4	C5	-0.3(4)	C9	C10	N2	C6	-2.4(3)
C3	C4	C5	C6	-179.9(2)	C9	C10	N2	Sc1	163.35(18)
C3	C4	C5	N1	-0.6(3)	N1	C1	C2	C3	0.4(4)
C4	C5	C6	C7	-12.2(3)	N1	C5	C6	C7	168.47(19)
C4	C5	C6	N2	168.7(2)	N1	C5	C6	N2	-10.7(3)
C4	C5	N1	C1	1.4(3)	N2	C6	C7	C8	-0.2(3)
C4	C5	N1	Sc1	-178.36(16)	O1	N3	O2	Sc1	5.99(19)
C5	C6	C7	C8	-179.3(2)	O2	N3	O1	Sc1	-5.94(19)
C5	C6	N2	C10	-178.37(18)	O3	N3	O1	Sc1	172.42(19)
C5	C6	N2	Sc1	15.6(2)	O3	N3	O2	Sc1	-172.38(18)
C6	C5	N1	C1	-179.23(18)	O5	N4	O6	Sc1	-0.44(19)
C6	C5	N1	Sc1	1.0(2)	O6	N4	O5	Sc1	0.44(19)
C6	C7	C8	C9	-2.3(4)	O7	N4	O5	Sc1	-178.86(19)
C7	C6	N2	C10	2.5(3)	O7	N4	O6	Sc1	178.85(19)

Table S8 Hydrogen Atom Coordinates (Å×10^4) and Isotropic Displacement Parameters (Å^2×10^3) for bath333n.

Atom	*x*	*y*	*z*	U(eq)
H1	-1039	3805	1328	37
H2	-1152	4184	-1040	43
H3	731	2254	-2402	48
H4	2673	-35	-1330	43
H7	3905	-2214	-96	42
H8	5689	-4392	1209	48
H9	6049	-4163	3339	45
H10	4465	-1838	4206	39
H4A	2070(40)	-910(40)	6120(40)	35(8)

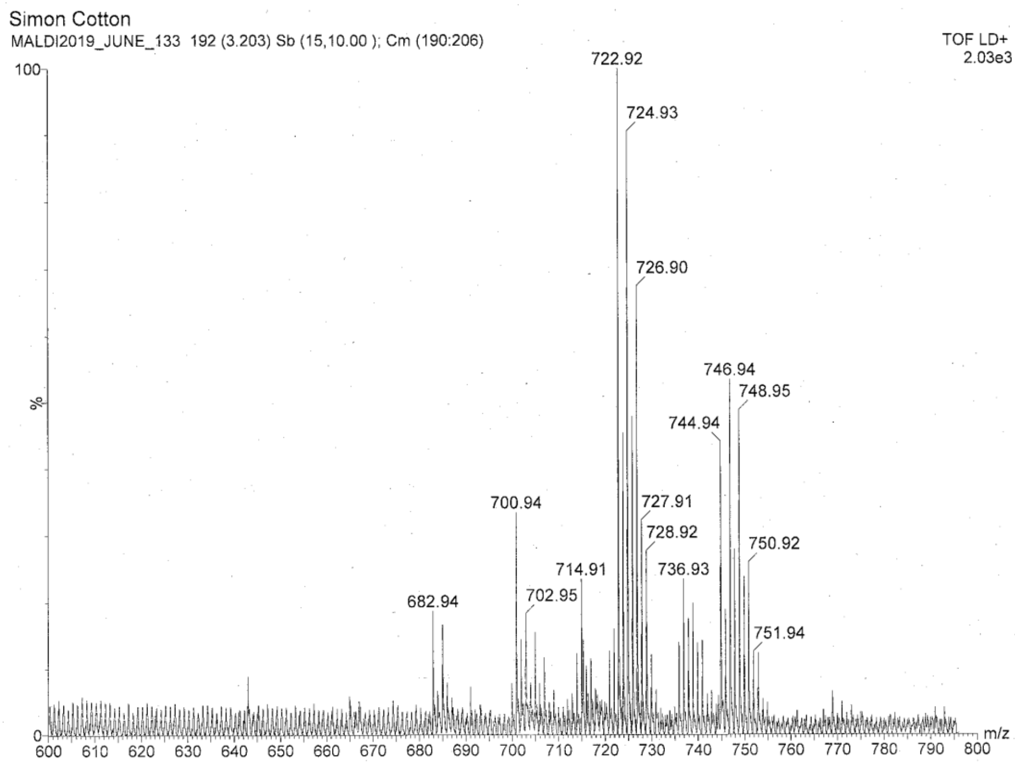

Figure S1 (a) MALDI Mass spectrum of [(bipy)$(NO_3)_2$Sc(μ-OH)$_2$Sc$(NO_3)_2$(bipy)].

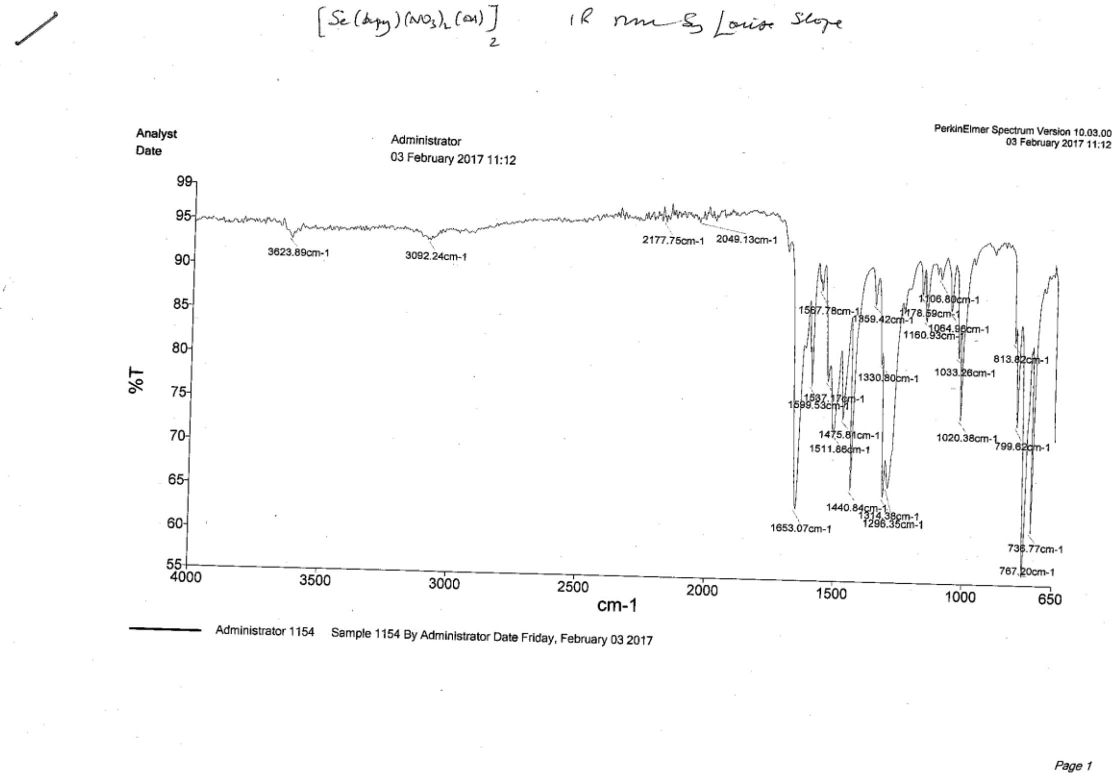

Figure S1 (b) Solid state IR spectrum of [(bipy)$(NO_3)_2$Sc(μ-OH)$_2$Sc$(NO_3)_2$(bipy)].

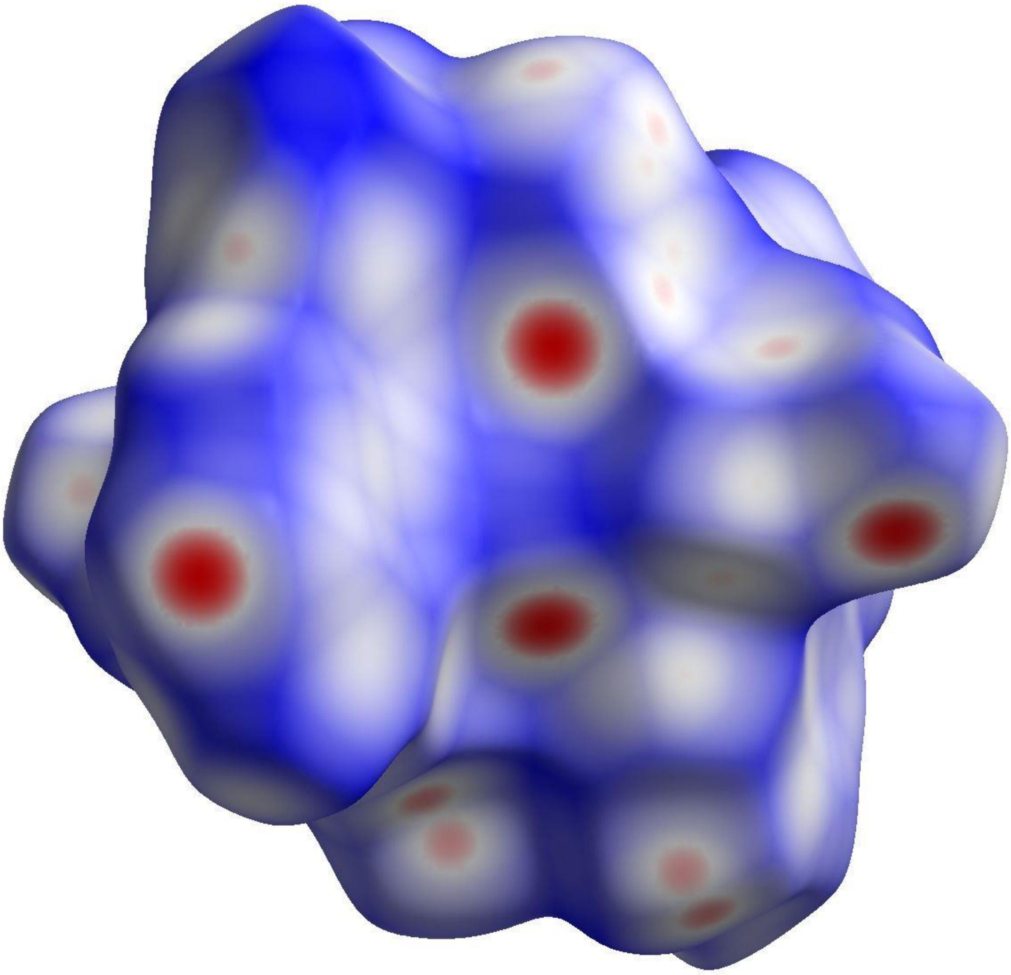

Figure S2. Surface plot of D-norm surface -0_3117_1_0733.

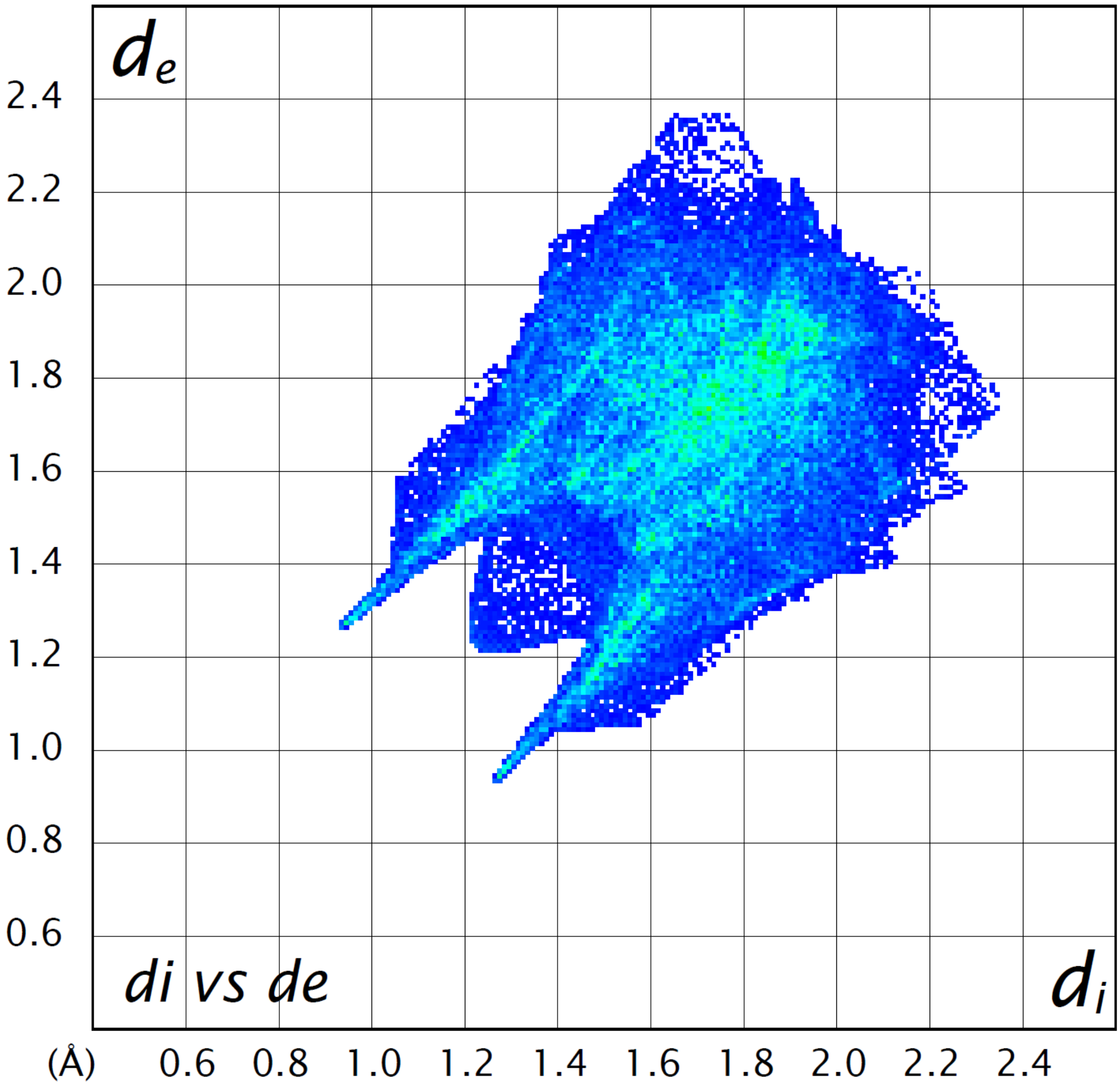

Figure S3. Fingerprint plot showing the presence of hydrogen bonding.

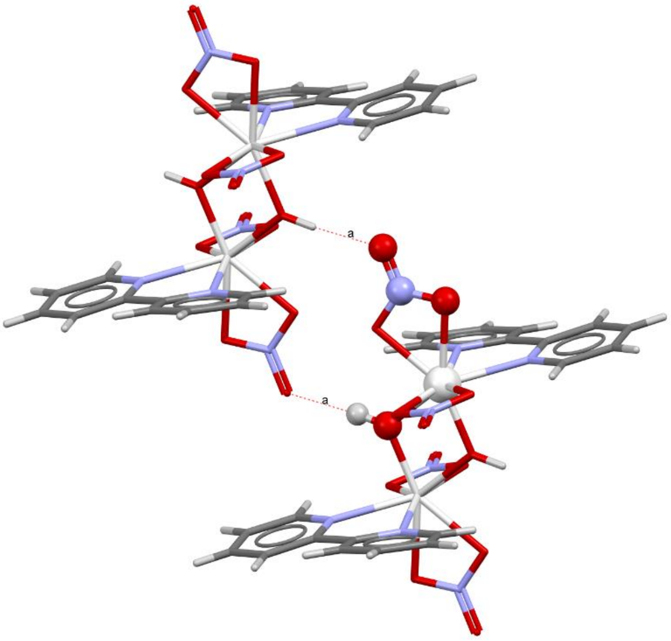

Figure S4. Packing plot showing the H-hydrogen bonding interactions and the presence of the R2,2(12) rings.
